# Clinico‐sero‐pathological profiles and risk prediction model of idiopathic inflammatory myopathy (IIM) patients with different perifascicular changes

**DOI:** 10.1111/cns.14882

**Published:** 2024-08-04

**Authors:** Lining Zhang, Lijun Fu, Guoyong Zhang, Ying Hou, Xiaotian Ma, Dandan Zhao, Wei Li, Tingjun Dai, Qiang Shu, Chuanzhu Yan, Bing Zhao

**Affiliations:** ^1^ Department of Rheumatology Qilu Hospital of Shandong University Jinan Shandong China; ^2^ Department of Neurology Qilu Hospital of Shandong University Jinan Shandong China; ^3^ School of Finance Southwestern University of Finance and Economics Chengdu China; ^4^ Department of Medicine Experimental Center, Qilu Hospital (Qingdao), Cheeloo College of Medicine Shandong University Qingdao Shandong China; ^5^ Shandong Key Laboratory of Medicine and Prevention Integration in Rheumatism and Immunity Disease Qilu Hospital of Shandong University Jinan Shandong China; ^6^ Department of Neurology, Qilu Hospital (Qingdao), Cheeloo College of Medicine Shandong University Qingdao Shandong China; ^7^ Research Institute of Neuromuscular and Neurodegenerative Diseases and Department of Neurology, Qilu Hospital Shandong University Jinan Shandong China; ^8^ Mitochondrial Medicine Laboratory, Qilu Hospital (Qingdao) Shandong University Qingdao Shandong China

**Keywords:** idiopathic inflammatory myopathy, perifascicular atrophy, perifascicular enhancement of MHC‐I and/or MHC‐II, perifascicular necrosis

## Abstract

**Aims:**

To explore the clinico‐sero‐pathological characteristics and risk prediction model of idiopathic inflammatory myopathy (IIM) patients with different muscular perifascicular (PF) changes.

**Methods:**

IIM patients in our center were enrolled and the clinico‐sero‐pathological data were retrospectively analyzed. A decision tree model was established through machine learning.

**Results:**

There were 231 IIM patients enrolled, including 53 with perifascicular atrophy (PFA), 39 with perifascicular necrosis (PFN), and 26 with isolated perifascicular enhancement of MHC‐I/MHC‐II (PF‐MHCn). Clinically, PFA patients exhibited skin rashes and dermatomyositis‐specific antibodies (DM‐MSAs, 74.5%) except for anti‐Mi2. PFN patients showed the most severe muscle weakness, highest creatine kinase (CK), anti‐Mi2 (56.8%), and anti‐Jo‐1 (24.3%) antibodies. PF‐MHCn patients demonstrated negative MSAs (48.0%) and elevated CK. Histopathologically, MAC predominantly deposited on PF capillaries in PFA but on non‐necrotic myofiber in PFN (43.4% and 36.8%, *p* < 0.001). MxA expression was least in PF‐MHCn (36.0% vs. 83.0% vs. 63.2%, *p* < 0.001). The decision tree model could effectively predict different subgroups, especially PFA and PFN.

**Conclusions:**

Three types of PF change of IIMs representing distinct clinico‐serological characteristics and pathomechanism. Undiscovered MSAs should be explored especially in PF‐MHCn patients. The three pathological features could be accurately predicted through the decision tree model.

## Graphical abstract

There are three kind of PF changes on muscle pathology of IIM patients, including PFA, PFN, and PF‐MHCn, each referring to different clinico‐sero‐pathological characteristics and distinct pathogenic mechanism. The tree‐based three‐stage clinico‐serological machine learning model could accurately predict which kind of PF changes an IIM patient would develop.

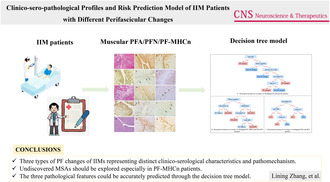

## 1 INTRODUCTION

Idiopathic inflammatory myopathies (IIMs) are a group of heterogeneous systemic autoimmune diseases, with organs such as skin, skeletal muscle and lung most often involved.^1^ The current recognized subgroups consist of dermatomyositis (DM), polymyositis (PM), immune‐mediated necrotizing myopathies (IMNM), antisynthetase syndrome (ASS), and sporadic inclusion body myositis (sIBM).^1^, ^2^, ^3^

Until now, more than 15 kinds of myositis‐specific autoantibodies (MSAs) were identified, and each of them seemed to be associated with distinct clinical and pathological manifestations.^1^ Furthermore, skeletal muscle pathology played an essential role in the diagnosis of IIMs. Among the variable pathological manifestations of IIMs, myofiber atrophy or necrosis, inflammatory infiltrates, or membrane attack complex (MAC) deposition on muscle fibers seemed indistinguishable from those mimicking muscle diseases such as muscular dystrophy.^3^, ^4^, ^5^, ^6^ However, the pathological changes in the perifascicular (PF) domains such as perifascicular atrophy (PFA), perifascicular necrosis (PFN) seemed to be characteristic in IIMs and related to some specific IIM subgroups.^7^

The concept of PFA was first proposed by Bohan & Peter as a diagnostic hallmark of DM and is still used today.^3^, ^8^, ^9^ The prevalence of PFA ranged from 37% to 51% in adult and approximately 80% in juvenile patients,^10^, ^11^ mainly associated with anti‐TIF1γ and anti‐NXP2 autoantibodies. However, the underlying mechanism remained unclear. The most frequently referred opinion was hypoxia in the PF area due to insufficient blood supply resulting from the damage of capillaries.^12^, ^13^ Recent years, the pathogenic role of type I interferon (IFN) for PFA was also increasingly proposed.^14^, ^15^

Otherwise, PFN was reported to occur predominantly in 28%–76% of patients with ASS,^16^, ^17^, ^18^ while it could also be observed in anti‐Mi2‐DM patients with a range of 30%–50%.^19^, ^20^ This pathological manifestation was deemed possibly due to changes in the perimysium, and these patients were classified as “immune myopathies with perimysial pathology.”^21^, ^22^

Furthermore, we could also observe a phenomenon that perifascicular enhancement of major histocompatibility complex (MHC)‐I and/or MHC‐II as the isolated PF change (PF‐MHCn) in some definite IIM patients.^18^ There was no morphological change such as necrosis or atrophy of the perifascicular muscle fibers, but the definite immunostaining of MHC‐I and/or MHC‐II suggested abnormal immunological events in this specific area.

To our knowledge, there are no systematic studies specifically focusing on the differences between patients with different PF pathologies, including PFA, PFN, and PF‐MHCn. The present study aimed to retrospectively summarize and compare the clinical, serological, myopathological characteristics as well as the long‐term prognosis among IIM patients with different PF changes. Furthermore, we attempted to establish a tree‐based machine learning model to predict different muscular PF changes based on the clinico‐serological features. Meanwhile, the underlying pathophysiological mechanisms were also discussed.

## 2 PATIENTS AND METHODS

### 2.1 Population

Consecutive patients who were diagnosed with IIMs both clinically and pathologically at our neuromuscular disease (NMD) center from January 2019 to July 2023 were enrolled in this study. The diagnosis and classification of PM, DM, and ASS was identified according to the Bohan & Peter criteria and Connors' criteria.^8^, ^9^, ^23^ IMNM and sIBM patients were excluded due to the fact that PF changes were scarcely observed in these two subgroups.^24^, ^25^ Finally, 231 patients were included in the present study.

Since this was a retrospective study, the required data including demographic information, clinical manifestations, and laboratory results were obtained according to the medical records. The prognosis, such as the visual analogue scale (VAS) and medication adherence, was obtained through outpatient or telephone follow‐up. In terms of clinical assessment, muscle strength was assessed by the ordinal 10‐point (0–10) manual muscle testing of 8 unilateral muscle groups (MMT8). Interstitial lung disease (ILD) was diagnosed on high resolution CT according to the recognized standards.^26^ Muscular and extra‐muscular disease activity were evaluated by physicians according to the 0–10 cm VAS.^27^ The Physicians' extra‐muscular VAS involved the comprehensive 10 cm‐VAS score of general condition, skin, osteoarticular, gastrointestinal tract, pulmonary, and cardiovascular systems. Relapse was confirmed according to disease flare criteria in IIMs.^28^

### 2.2 MSAs detection

All patients' serum samples were collected and stored at −80°C in our NMD center. Dot immunoassay (Autoimmune Myositis Profile Antibody IgG Detection Kit, MyBiotech Co., Ltd, Xi'an, China, MT559) was used following the manufacturer's instructions for MSAs detection, including DM specific antibodies (anti‐Mi2, anti‐ MDA5, anti‐TIF1γ, anti‐NXP2, anti‐SAE), anti‐aminoacyl tRNA synthetase (ARS) antibodies (anti‐Jo‐1, anti‐PL‐7, anti‐PL‐12, anti‐EJ, anti‐OJ, anti‐KS, and anti‐Zo, anti‐Ha), anti‐SRP, anti‐HMGCR, and anti‐cN1A antibodies.

### 2.3 Muscle histopathological evaluation

All enrolled IIM patients underwent an open muscle biopsy for routine diagnostic purposes. Seven‐micrometer cryostat sections were stained for hematoxylin and eosin (HE), succinate dehydrogenase (SDH), cytochrome oxidase (COX), anti‐MHC‐I rabbit monoclonal antibody (clone EP1395Y; Abcam), anti‐MHC‐II mouse monoclonal antibody (clone CR3/43; Dako), anti‐C5b‐9 (MAC) mouse monoclonal antibody (clone aE11; Dako), and anti‐MxA rabbit polyclonal antibody (ab95926; Abcam), anti‐CD3 mouse monoclonal antibody (clone LN10; Zhongshan Golden Bridge Biotechnology), anti‐CD20 rabbit monoclonal antibody (ab78237; Abcam).

According to previous studies, the term “perifascicular changes” describes alterations of the myofibers along any edge of the muscle fascicles.^7^ PFA was documented when more than 60% of fibers were atrophic along one edge of a fascicle.^7^, ^29^ PFN was defined as more than two‐thirds of necrotic fibers presenting in the perifascicular region.^16^, ^20^ PF‐MHCn indicated isolated MHC‐I and/or MHC‐II expression in the PF region or diffuse expression with enhancement in the PF region, without PFA or PFN.

The juvenile DM scoring system was applied with slight modifications in the evaluation of atrophic or necrotic myofibers, CD3^+^ and CD20^+^ lymphocytes.^7^, ^29^ Myofiber expression of MHC‐I and/or MHC‐II was defined as sarcolemmal staining combined with or without sarcoplasmic staining. MxA expression on non‐necrotic myofiber sarcoplasm and MAC deposition on non‐necrotic sarcolemma or capillaries were recorded, respectively.

### 2.4 Standard protocol approvals and patient consents

This study was approved by the Ethics Committee of Qilu Hospital (Qingdao), Shandong University, China (KYLL‐KS‐2022054). Consents for tests and publication were obtained from all the patients or their parents in the present study. The study was performed in accordance with the Declaration of Helsinki.

### 2.5 Statistical analysis

SPSS version 26.0 (SPSS, Inc., Chicago, IL, USA) was used for statistical analysis. Qualitative data are described as frequencies (percentages), while quantitative variables are expressed as mean ± standard deviation or median and interquartile range according to whether the data conform to the normal distribution or not. To compare qualitative data, chi‐squared test or Fisher's exact test was used. *T*‐test for normal distributed data, Mann–Whitney *U*‐test or Kruskal–Wallis *H* test for non‐normal distributed data were performed to compare means or medians among groups. Multivariate logistic regression analysis was performed to define risk factors for muscular PF changes. *p* < 0.05 were considered statistically significant.

A tree‐based three‐stage machine learning model was used to enhance muscular PF changes prediction. Three classification stages followed the same machine learning framework, which could be regarded as the logistic regression‐decision tree (LR‐DT) model. Under this model framework, a specific logistic regression model was first established to identify risk factors, which were then included in the following decision tree to obtain a concise but effective classification model for predicting muscular PF changes. The optimal decision tree for each stage was selected by the trade‐off between goodness of fit and prediction performance. Additionally, the prediction power was evaluated by 10‐fold cross‐validation method. All algorithms are calculated by utilizing the framework of Python 3.9's scientific computing package, namely sklearn. More detailed procedures can be found in Data S1 (including Figure S1 and Table S1).

## 3 RESULTS

### 3.1 Risk factors for IIM patients presented with PF changes on muscle pathology

There was a total of 231 IIM patients enrolled in the present study, including 118 with PF changes (PF group) and 113 without PF changes (non‐PF group). The univariate analysis of possible risk factors associated with PF changes was shown in Tables S2 and S3. As a result, skin involvement, Heliotrope rash, MMT8, anti‐TIF1γ antibodies, anti‐Mi2 antibodies, MSAs‐negative, punched‐out fibers, MAC deposited on capillaries, and decreased/absent COX activity were included for further multivariate analysis (Table 1). Finally, the analysis identified the presence of anti‐Mi2 antibodies [odds ratio (OR) = 5.622, 95% CI (1.562, 20.231), *p* = 0.008], punched‐out fibers [OR = 9.684, 95% CI (3.668, 25.569), *p* < 0.001], MAC deposited on capillaries [OR = 2.398, 95% CI (1.172, 4.908), *p* = 0.017], and decreased/absent COX activity [OR = 2.112, 95% CI (1.092, 4.084), *p* = 0.026] as independent risk factors associated with muscular PF changes. By contrast, negative MSA protected the patients from perifascicular involvement [OR = 0.451, 95% CI (0.208, 0.978), *p* = 0.044].

**TABLE 1:** Multivariate analysis of the risk factors associated with muscular PF changes.

Variables	OR (95% CI)	*p*‐Value
Skin involvement, *n* (%)	1.676 (0.793, 3.539)	0.176
Heliotrope rash, *n* (%)	0.792 (0.318, 1.974)	0.617
MMT8, median (IQR)	0.987 (0.958, 1.017)	0.389
Anti‐TIF1γ antibodies, *n* (%)	1.101 (0.363, 3.341)	0.865
Anti‐Mi2 antibodies, *n* (%)	5.622 (1.562, 20.231)	0.008*
MSAs‐negative, *n* (%)	0.451 (0.208, 0.978)	0.044*
Punched‐out fibers, *n* (%)	9.684 (3.668, 25.569)	<0.001*
MAC deposited on capillaries, *n* (%)	2.398 (1.172, 4.908)	0.017*
Decreased/absent COX activity, *n* (%)	2.112 (1.092, 4.084)	0.026*

*Note*: *p < 0.05.

Abbreviations: CI, confidence interval; COX, cytochrome oxidase; MAC, membrane attack complex; MMT, manual muscle testing; MSAs, myositis‐specific autoantibodies; OR, odds ratio; PF, perifascicular area.

### 3.2 Comparison of clinico‐serological profiles among PFA, PFN and PF‐MHCn subgroups

Among the 118 IIM patients with PF changes, 53 were identified with PFA, 39 with PFN and 26 with PF‐MHCn. To better clarify the patients with different PF changes, we compared the clinico‐sero‐pathological characteristics among the three subgroups. In particular, juvenile IIM patients were more likely to exhibit PFA, followed by PF‐MHCn, but not PFN (32.1% vs. 11.5% vs. 0, *p* < 0.001), which directly decreased the median age of PFA subgroup (40 vs. 58 vs. 57, *p* < 0.001). DM specific skin rashes, such as Gottron's sign/papules and Heliotrope rash were more common in patients with PFA compared with the other two subgroups (86.8% vs. 59.0% vs. 69.2%, *p* = 0.009). In addition, patients with PFN achieved the lowest MMT8 and highest serum CK levels (62 vs. 65 vs. 69, *p* = 0.009; 3180 vs. 687 vs. 219, *p* < 0.001). Interestingly, PF‐MHCn patients featured the mildest muscle weakness, but had higher CK levels than the PFA patients, which is inconsistent with our previous perception. There was no obvious difference in other clinical manifestations among the three subgroups.

**FIGURE 1:**
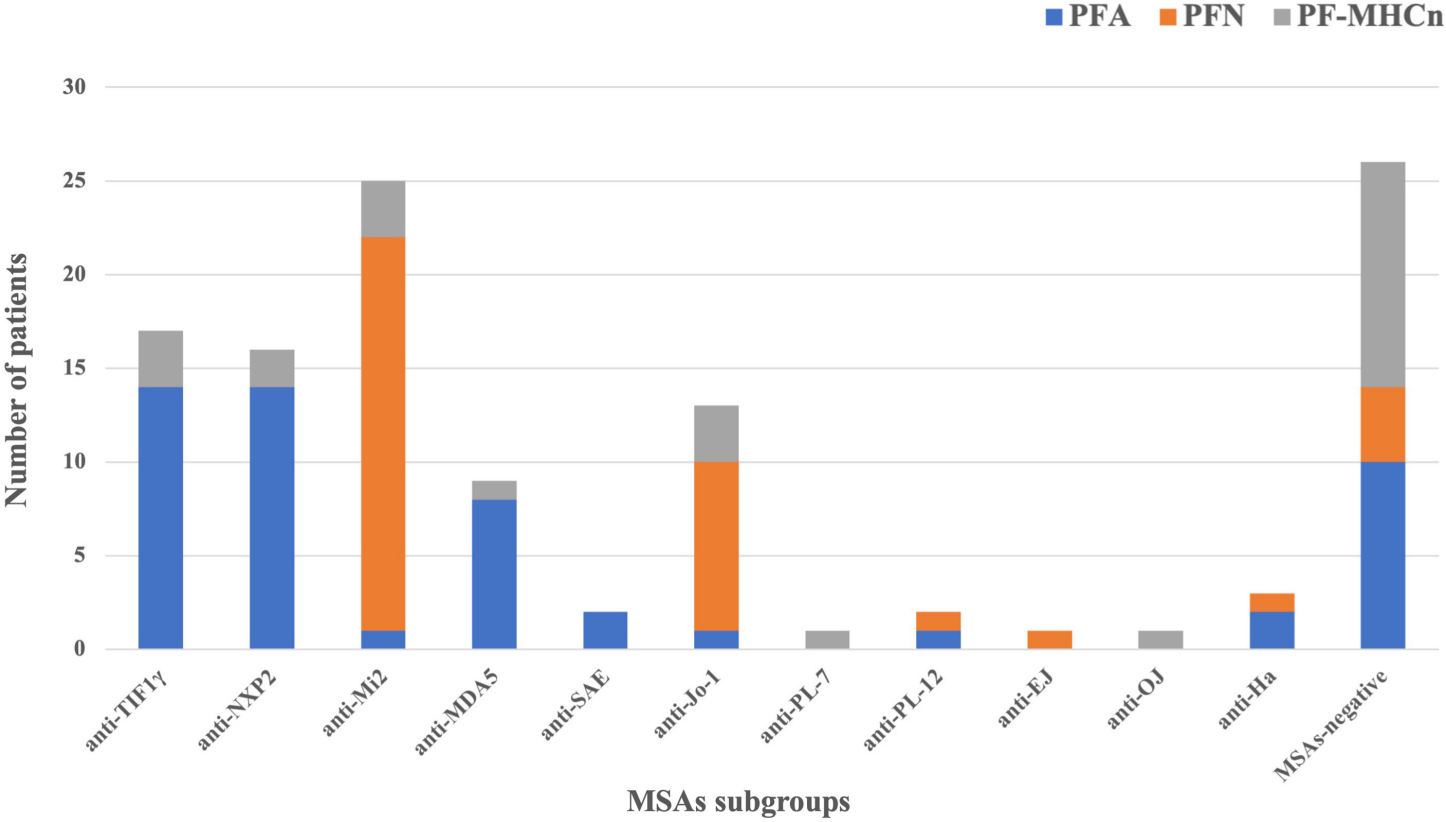
MSAs distributions among the three muscular PF subgroups. MHC, major histocompatibility complex; MSAs, myositis‐specific autoantibodies; PFA, perifascicular atrophy; PF‐MHCn, perifascicular enhancement of MHC‐I and/or MHC‐II; PFN, perifascicular necrosis.

Regarding MSAs profiles, the most prevalent MSAs in PFA subgroup were anti‐TIF1γ, anti‐NXP2, and anti‐MDA5 antibodies (27.5%, 27.5%, and 15.7% respectively), all of which were DM‐MSAs and higher than the other two subgroups (*p* all <0.001) (Table 2 and Figure 1). Besides, 7.9% of PFA patients were found to be positive for anti‐ARS antibodies, and 19.6% were seronegative. In addition, anti‐Mi2 and anti‐Jo‐1 antibodies were much more prevalent in PFN than in the other two groups (56.8% vs. 12.0% vs. 2.0%, *p* < 0.001; 24.3% vs. 12.0% vs. 2.0%, *p* = 0.003). In patients with isolated PF‐MHCn, approximately half of them were MSAs‐negative, significantly different from the other two subgroups (48.0% vs. 19.6% vs. 10.8%, *p* = 0.002). The remaining PF‐MHCn patients were positive for various MSAs including 32.0% DM‐MSAs and 20.0% anti‐ARS antibodies.

**TABLE 2:** Comparison of clinico‐serological characteristics among groups with different perifascicular changes.

	PFA (*n* = 53)	PFN (*n* = 39)	PF‐MHCn (*n* = 26)	*p*‐Value
Female, *n* (%)	32 (60.4)	27 (69.2)	14 (53.8)	0.437
Age of onset, median (IQR) years	40 (12,56)	58 (49,67)	57 (44,68)	<0.001*
Juvenile,^a^ *n* (%)	17 (32.1)	0 (0)	3 (11.5)	<0.001*
Disease duration,^b^ median (IQR) months	5 (3,12)	3 (2,8)	3 (1,8)	0.121
Skin involvement, *n* (%)	46 (86.8)	23 (59.0)	18 (69.2)	0.009*
Gottron's sign/papules	21 (39.6)	11 (28.2)	3 (11.5)	0.036*
Heliotrope rash	23 (43.4)	10 (25.6)	3 (11.5)	0.011*
Muscle weakness, *n* (%)	47 (88.7)	38 (97.4)	23 (88.5)	0.290
Myalgia, *n* (%)	29 (54.7)	17 (43.6)	13 (50.0)	0.573
Arthralgia, *n* (%)	13 (24.5)	11 (28.2)	6 (23.1)	0.879
Dysphagia, *n* (%)	21 (36.9)	20 (51.3)	6 (23.1)	0.075
ILD,^c^ *n* (%)	19 (37.3)	18 (48.6)	11 (44.0)	0.557
Malignancy, *n* (%)	5 (9.4)	4 (10.3)	3 (11.5)	>0.999
MMT8, median (IQR)	65 (57,74)	62 (49,70)	69 (63,77)	0.009*
CK at muscle biopsy, median (IQR) U/L	219 (99,482)	3180 (1228,4848)	687 (285,2922)	<0.001*
ANA positive,^d^ *n* (%)	28 (75.7)	29 (96.7)	15 (75.0)	0.022*
MSAs,^c^ *n* (%)				
DM‐MSAs	38 (74.5)	21 (56.8)	8 (32.0)	0.001*
Anti‐TIF1γ	14 (27.5)	0 (0)	3 (12.0)	<0.001*
Anti‐NXP2	14 (27.5)	0 (0)	2 (8.0)	<0.001*
Anti‐Mi2	1 (2.0)	21 (56.8)	3 (12.0)	<0.001*
Anti‐MDA5	8 (15.7)	0 (0)	1 (4.0)	0.017*
Anti‐SAE	2 (3.9)	0 (0)	0 (0)	0.702
Anti‐ARS	4 (7.9)	12 (32.4)	5 (20.0)	0.012*
Anti‐Jo‐1	1 (2.0)	9 (24.3)	3 (12.0)	0.003*
Anti‐non‐Jo‐1 ARS	3 (5.7)	3 (7.7)	2 (7.7)	0.904
Anti‐PL‐7	0 (0)	0 (0)	1 (4.0)	0.221
Anti‐PL‐12	1 (2.0)	1 (2.7)	0 (0)	>0.999
Anti‐EJ	0 (0)	1 (2.7)	0 (0)	0.549
Anti‐OJ	0 (0)	0 (0)	1 (4.0)	0.221
Anti‐Ha	2 (3.9)	1 (2.7)	0 (0)	>0.999
MSAs‐negative	10 (19.6)	4 (10.8)	12 (48.0)	0.002*

*Note*: *p < 0.05.

Abbreviations: ANA, antinuclear antibody; ARS, aminoacyl tRNA synthetase; CK, creatine kinase; DM, dermatomyositis; ILD, interstitial lung disease; IQR, interquartile range; MHC, major histocompatibility complex; MMT, manual muscle testing; MSAs, myositis‐specific autoantibodies; PFA, perifascicular atrophy; PF‐MHCn, perifascicular enhancement of MHC‐I and/or MHC‐II; PFN, perifascicular necrosis.

^a^ Age ≤ 18 years.

^b^ The time from the first myositis symptom to the diagnosis of IIM.

^c^ *N* = 113 (51 + 37 + 25).

^d^ *N* = 87 (37 + 30 + 20).

### 3.3 Comparison of histopathological characteristics among PFA, PFN and PF‐MHCn subgroups

**FIGURE 2:**
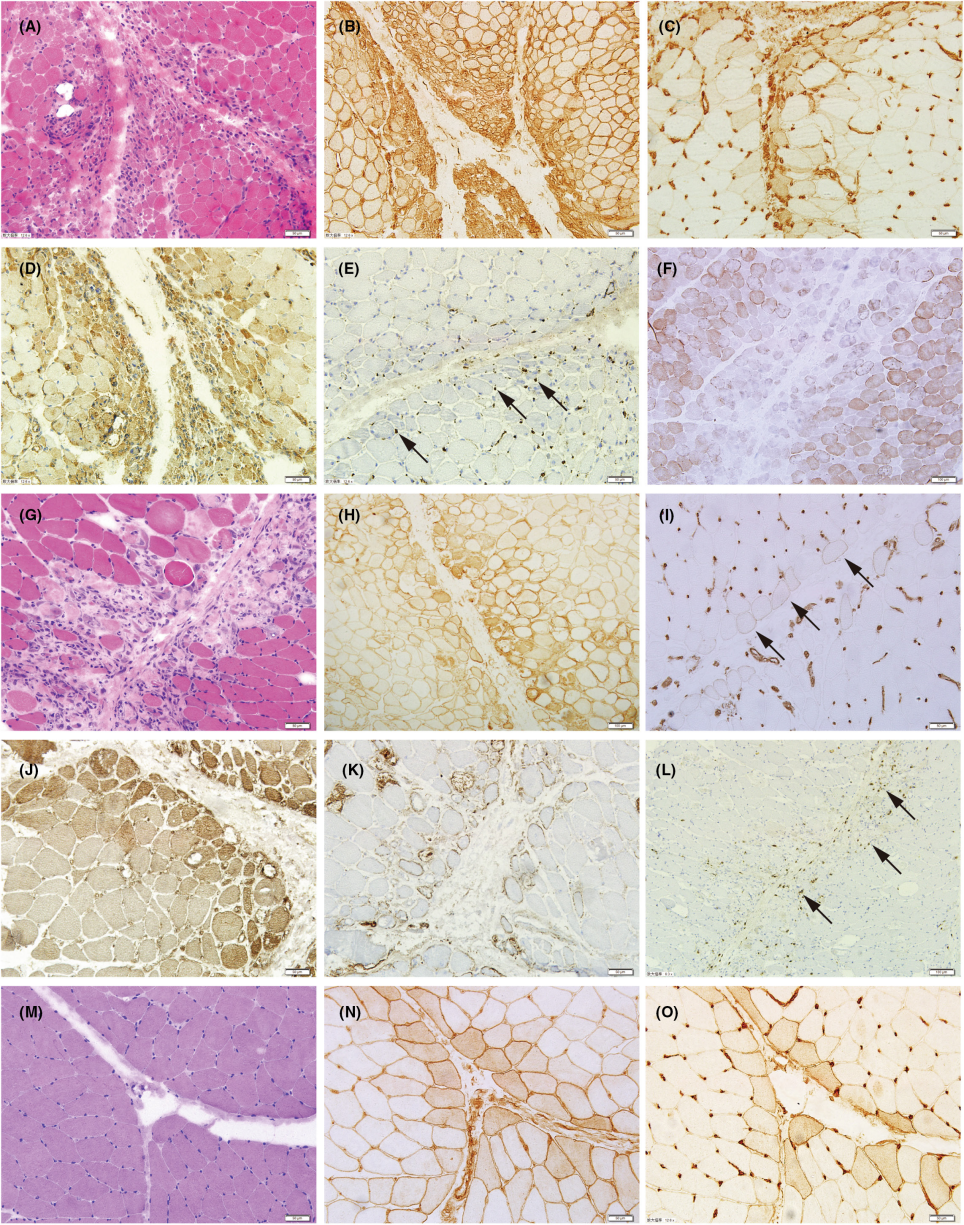
Representative histopathologic figures of the three subgroups. A–F, PFA: (A) Typical PFA with punch‐out fibers adjacent to the wide perimysium (HE staining). (B) A gradient MHC‐I upregulation from periphery toward the centrofascicle. (C) MHC‐II expression only in the PF region. (D) Myofiber MxA expression in the PF region and the adjacent centrofascicle. (E) Prominent MAC deposition on capillaries alongside the PF edge (arrows). (F) Multiple blue fibers in the PF region (COX/SDH double staining). G–L, PFN: (G) Typical PFN with massive inflammatory infiltration in endomysium along the PF region (HE staining). (H) A gradient MHC‐I upregulation from periphery toward the centrofascicle. (I) Perifascicular MHC‐II expression in scattered myofibers in the PF region (arrows). (J) Diffuse MxA expression reinforced in the PF region. (K) Prominent MAC deposition on non‐necrotic myofiber sarcolemma alongside the PF edge. (L) Evident lymphocytes infiltration in endomysium alongside the PF edge (anti‐CD3 staining, arrows). M–O, PF‐MHCn: (M) No morphological change of muscle fibers along the PF region could be observed (HE staining), while MHC‐I (N) and MHC‐II (O) were definitely upregulated in the PF myofibers on consecutive sections. COX, cytochrome oxidase; MAC, membrane attack complex; MHC, major histocompatibility complex; MxA, myxovirus resistance protein; PF, perifascicular; PFA, perifascicular atrophy; PF‐MHCn, perifascicular enhancement of MHC‐I and/or MHC‐II; PFN, perifascicular necrosis; SDH, succinate dehydrogenase.

The detailed histopathological features of these three subgroups were summarized in Table 3 and Figure 2. The myofiber necrosis score was highest in patients with PFN (2 vs. 1 vs. 1, *p* < 0.001). Despite this, more than half of the patients with PFA or PF‐MHCn also showed scattered myofiber necrosis in the centrofascicular region. Punched‐out fibers were more common seen in PFA patients (69.8% vs. 35.9% vs. 15.4%, *p* < 0.001) (Figure 2A). MHC‐I and MHC‐II expression could be identified in the majority of all three subgroups and most of them showed a pattern of PF enhancement (Table 3, Figure 2B,C,H,I,N,O, and Figure S2). In contrast, sarcoplasmic MxA expression was observed in 83.0% of PFA patients, 63.2% of PFN patients but only 36.0% of PF‐MHCn patients (*p* < 0.001), also with the PF enhancement being the most common pattern (Figure 2D,J). In addition, the pattern of MAC deposition was significantly different between PFA and PFN subgroups: MAC deposition on capillaries was most commonly identified in the PFA subgroup (69.8%), with 43.4% showing a PF pattern (*p* < 0.001, Figure 2E), while MAC deposition on non‐necrotic myofibers was most commonly observed in the PFN subgroup (84.2%), with 36.8% exhibiting a PF pattern (*p* = 0.004) (Figure 2K). Regarding the inflammatory infiltration, patients with PFN showed the highest endomysial inflammatory score, especially in the endomysium along the PF region [1(1,2) vs. 1(0,1) vs. 1(0,1), *p* < 0.001, Figure 2L], which might be related to the most severe muscle damage in this subgroup. Furthermore, decreased/absent COX activity was more likely to be observed in the PFA patients compared to the other two subgroups (77.4% vs. 61.5% vs. 50.0%, *p* = 0.041, Figure 2F).

**TABLE 3:** Comparison of muscular histopathologic characteristics among groups with different perifascicular changes.

	PFA (*n* = 53)	PFN (*n* = 39)	PF‐MHCn (*n* = 26)	*p*‐Value
Myofiber abnormalities				
Myofiber necrosis score, median (IQR)	1 (0,1)	2 (2,2)	1 (0,2)	<0.001*
Myofiber necrosis, *n* (%)	28 (52.8)	39 (100)	14 (53.8)	<0.001*
Punched‐out fibers, *n* (%)	37 (69.8)	14 (35.9)	4 (15.4)	<0.001*
MHC‐I expression, *n* (%)				
Positive expression	53 (100)	39 (100)	26 (100)	/
Diffuse expression only	4 (7.5)	10 (25.6)	2 (7.7)	0.027*
PF enhancement	49 (92.5)	29 (74.4)	23 (88.5)	0.045*
MHC‐II expression^a^, *n* (%)				
Positive expression	39 (73.6)	27 (71.1)	18 (72.0)	0.964
Diffuse expression only	0 (0)	4 (10.5)	0 (0)	0.012*
PF enhancement	35 (66.0)	21 (55.3)	17 (68.0)	0.484
MxA expression,^a^ *n* (%)				
Positive expression	44 (83.0)	24 (63.2)	9 (36.0)	<0.001*
Diffuse expression only	2 (3.8)	0 (0)	0 (0)	0.298
PF enhancement	39 (73.6)	23 (60.5)	9 (36.0)	0.006*
MAC deposition,^a^ *n* (%)				
On non‐necrotic sarcolemma	19 (35.8)	32 (84.2)	9 (36.0)	<0.001*
PF pattern	6 (11.3)	14 (36.8)	2 (8.0)	0.004*
On capillaries	37 (69.8)	17 (44.7)	14 (56.0)	0.054
PF pattern	23 (43.4)	5 (13.2)	2 (8.0)	<0.001*
Inflammatory infiltration score,^b^ median (IQR)				
Total score	2 (1,5)	3 (2,5)	1 (1,3)	0.094
Perimysial score	1 (0,2)	1 (0,2)	0 (0,1)	0.478
Endomysial score	1 (0,1)	1 (1,2)	1 (0,1)	<0.001*
Perivascular score	1 (0,2)	1 (0,1)	1 (0,2)	0.779
Histochemical staining, *n* (%)				
Decreased/absent COX activity	41 (77.4)	24 (61.5)	13 (50.0)	0.041*
Blue fiber on COX/SDH	31 (58.5)	15 (38.5)	10 (38.5)	0.096

*Note*: *p < 0.05.

Abbreviations: COX, cytochrome oxidase; IQR, interquartile range; MAC, membrane attack complex; MHC, major histocompatibility complex; MxA, myxovirus resistance protein; PFA, perifascicular atrophy; PF‐MHCn, perifascicular enhancement of MHC‐I and/or MHC‐II; PFN, perifascicular necrosis; SDH, succinate dehydrogenase.

^a^ *N* = 116 (53 + 38 + 25).

^b^ Total score of CD3 and CD20 lymphocyte: <4cells/20HPF = 0; 4–10 (1 cluster)/20HPF = 1; ≥2 clusters or >20cells/20HPF = 2.

### 3.4 Prediction of pathological subgroups using a three‐stage LR‐DT model

**FIGURE 3:**
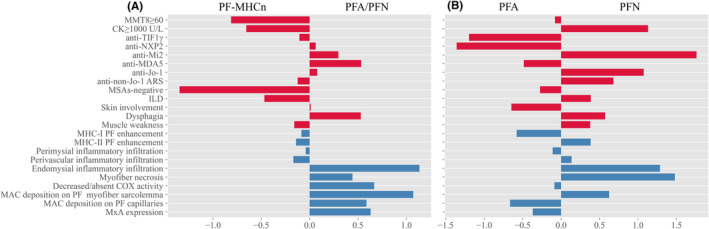
Multivariate analysis of logistic ridge regression for Stage 2 (A) and Stage 3 (B). ARS, anti‐aminoacyl tRNA synthetase; CK, creatine kinase; COX, cytochrome oxidase; ILD, interstitial lung disease; MAC, membrane attack complex; MMT, manual muscle testing; MSAs, myositis‐specific autoantibodies; MxA, myxovirus resistance protein; PFA, perifascicular atrophy; PF‐MHCn, perifascicular enhancement of MHC‐I and/or MHC‐II; PFN, perifascicular necrosis; SDH, succinate dehydrogenase.

To easily position one patient in these different classification subgroups before or without a muscle biopsy, a three‐stage LR‐DT model was used to establish a comprehensive prediction framework. First, we applied a specific logistic ridge regression to identify key clinico‐sero‐pathological factors that differentiate between PF and non‐PF subgroups (Table 1), PF‐MHCn and “PFA/PFN” subgroups, as well as PFA and PFN subgroups (Figure 3). As Table 1 showed, clinico‐serologically significant risk factor and protective factor for PF changes were anti‐Mi2 antibodies and MSAs‐negative, respectively. Furthermore, compared to “PFA/PFN,” the strong clinico‐serological factors associated with PF‐MHCn were MSAs‐negative, MMT8 ≥ 60 and CK ≥ 1000 U/L (Figure 3A). Additionally, the evident clinico‐serological factors correlated with PFA were anti‐TIF1γ and anti‐NXP2 antibodies, while those for PFN were anti‐Mi2, anti‐Jo‐1 antibodies and CK ≥ 1000 U/L (Figure 3B).

**FIGURE 4:**
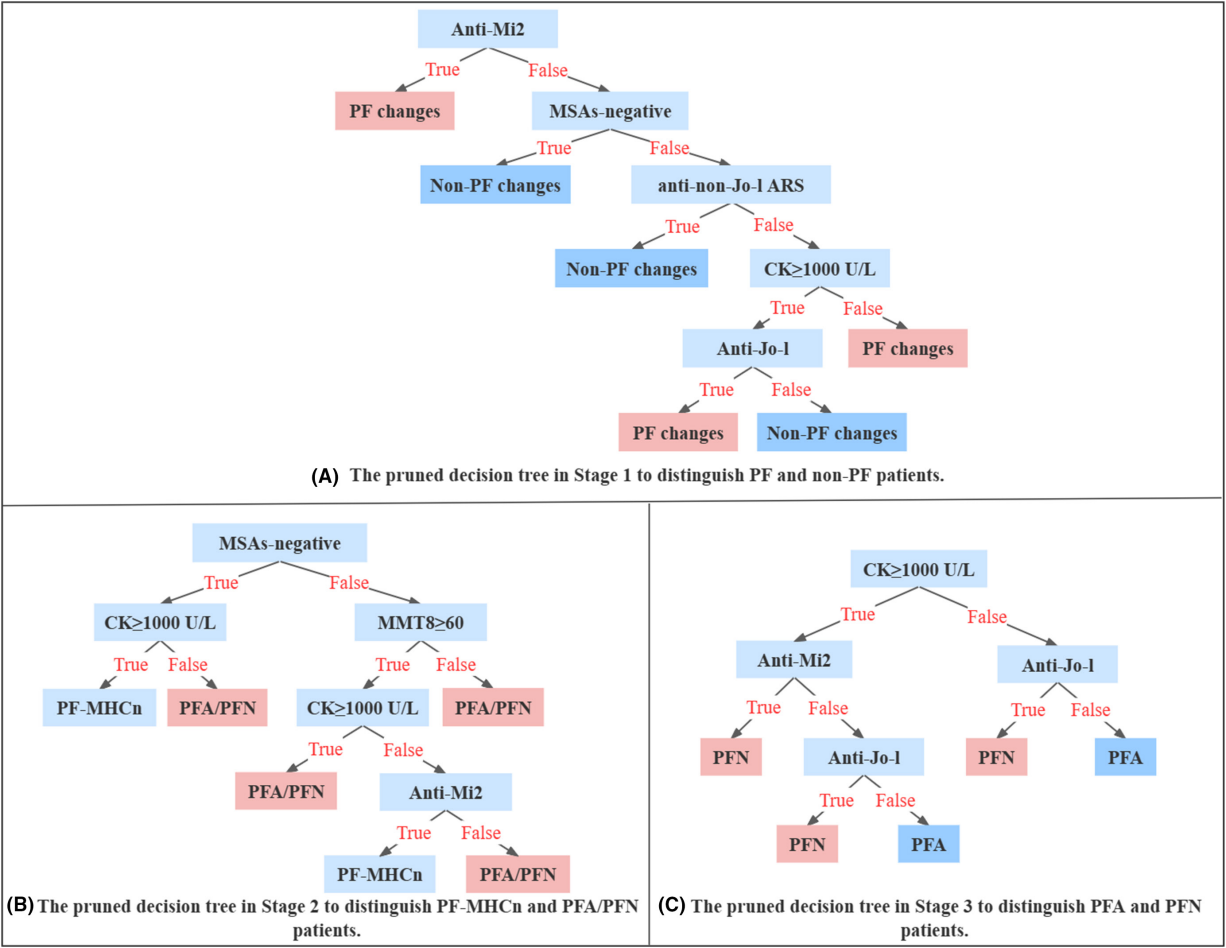
The three‐stage pruned prediction model with the framework of three decision trees. (A) Decision tree to distinguish PF and non‐PF changes patients; (B) Decision tree to distinguish PF‐MHCn and “PFA/PFN” patients; (C) Decision tree to distinguish PFA and PFN patients. ARS, anti‐aminoacyl tRNA synthetase; CK, creatine kinase; MHC, major histocompatibility complex; MMT, manual muscle testing; MSAs, myositis‐specific autoantibodies; PFA, perifascicular atrophy; PF‐MHCn, perifascicular enhancement of MHC‐I and/or MHC‐II; PFN, perifascicular necrosis.

After that, based on the identification results of clinico‐serological factors, three decision trees were established to predict the muscle pathologic change of a newly diagnosed IIM patient. To avoid overfitting and simplify the model, only critical variables were retained after pruning these three decision trees, since there was no further significant improvement in terms of accuracy with the addition of any more variables (Table S4). The final three decision trees of the three stages were presented in Figure 4. In detail, decision tree in Stage 1 was established by anti‐Mi2, MSAs‐negative, anti‐non‐Jo‐1 ARS, CK ≥ 1000 U/L, and anti‐Jo‐1 antibodies (Figure 4A). Decision tree in Stage 2 was performed by four clinico‐serological indicators: MSAs‐negative, CK ≥ 1000 U/L, MMT8 ≥ 60, anti‐Mi2 antibodies (Figure 4B). For Decision tree in Stage 3, CK ≥ 1000 U/L, anti‐Mi2, anti‐Jo‐1 antibodies were included to construct the prediction model (Figure 4C). The cross validation AUCs for Stage 1, Stage 2, and Stage 3 were 0.708, 0.769, and 0.872, respectively, which indicated that the proposed methods can effectively address the PFA and PFN prediction (Table S4).

### 3.5 Prognosis and immunotherapy of patients with PF changes at last follow‐up

As 23 patients were lost to contact, there were finally 95 patients included for long‐term follow‐up with a median time of 15 months (Table 4). Most of the patients could achieve a normal CK level at their last follow‐up. Improvement could be identified in both physicians' muscular and extra‐muscular VAS of all patients during follow‐up. Although the most severe muscular VAS in PFN patients [5(5,6) vs. 4(3,5) vs. 4(2,5), *p* < 0.001], they had the highest probability of reaching medication discontinuation at last follow‐up (19.4% vs. 5.0% vs. 0, *p* = 0.026). Moreover, there were eight patients who died of IIMs, with four, three, and one in each subgroup, respectively.

**TABLE 4:** Prognosis and immunotherapy of the three subgroups at the time of last follow‐up.

	PFA (*n* = 40)	PFN (*n* = 31)	PF‐MHCn (*n* = 24)	*p*‐Value
Follow up time, median (IQR) months	15 (6,33)	15 (6,31)	15 (5,39)	0.871
Normal CK,^a^ *n* (%)	32 (80)	18 (60)	17 (73.9)	0.177
Physicians' muscular VAS, median (IQR)				
At biopsy	4 (3,5)	5 (5,6)	4 (2,5)	<0.001*
At last follow‐up	1 (0,2)	2 (1,3)	2 (0,2)	0.048*
Physicians' extra‐muscular VAS, median (IQR)				
At biopsy	4 (2,5)	3 (2,4)	3 (2,4)	0.864
At last follow‐up	1 (0,2)	1 (1,3)	1 (0,2)	0.799
Therapy at last follow‐up, *n* (%)				
Pred use	38 (95.0)	25 (80.6)	24 (100)	0.026*
IS use	27 (67.5)	19 (61.3)	16 (66.7)	0.085
Drug‐free	2 (5.0)	6 (19.4)	0 (0)	0.026*
Relapse	13 (32.5)	8 (25.8)	4 (16.7)	0.378
Treatment course before relapse, median (IQR) months	14 (6,36)	19 (4,36)	26 (7,45)	0.871
Pred dosage when relapse, *n* (%)	15 (6,20)	15 (5,24)	16 (11,24)	0.917
Death, *n* (%)	4/44 (9.1)	3/34 (8.8)	1/25 (7.8)	0.804

*Note*: *p < 0.05.

Abbreviations: CK, creatine kinase; IQR, interquartile range; IS, immunosuppressant; MHC, major histocompatibility complex; MMT, manual muscle testing; PFA, perifascicular atrophy; PF‐MHCn, perifascicular enhancement of MHC‐I and/or MHC‐II; PFN, perifascicular necrosis; Pred, prednisone.

^a^ *N* = 93 (40 + 30 + 23).

## 4 DISCUSSION

In the present study, we summarized and compared the independent characteristics of IIM patients with three different types of PF changes. Specifically, the PFA patients mainly carried DM‐MSAs except for anti‐Mi2 antibodies, with skin rashes, moderate muscle involvement, MAC deposition on capillaries, more punched‐out fibers, and decreased/absent COX activity on muscle pathology. By contrast, the PFN patients always presented with anti‐Mi2 and anti‐Jo‐1 antibodies, higher CK levels, the most severe muscle damage, MAC deposition on non‐necrotic sarcolemma, and inflammatory infiltration along the PF region. Juvenile IIM patients were most likely to exhibit PFA, while none of them showed PFN. What' new, most patients with PF‐MHCn were MSAs‐negative and had moderately elevated CK levels, probably due to the easily observed necrotic myofibers scattered in the centrofascicle. Despite the most severe muscle damage, the PFN individuals were more likely to achieve drug‐free status. Consistently, supervised machine learning also revealed the distinct characteristics for each of the PF subgroup. The three‐stage LR‐DT model in this study based on clinico‐serological indicators could accurately predict whether an IIM patient would develop PF changes on muscle pathology, and then specifically position the patients with PF changes into one of the three subgroups.

PFA was usually recognized as the hallmark of DM patients.^11^, ^30^ In our cohort, 74.5% of the patients with PFA carried DM‐MSAs including anti‐TIF1γ, anti‐NXP2, anti‐MDA5, and anti‐SAE antibodies. Consistently, skin rashes especially the Gottron's signs/papules and Heliotrope rashes were the characteristics of this group. What's more, PFA was also observed in 19.0% (4/21) of the patients with anti‐ARS antibodies, suggesting that PFA was not completely specific to DM. This was consistent with previous studies, which reported the prevalence of PFA ranging from 17.0% to 44.4% in ASS.^20^, ^31^ Most of the juvenile patients in our cohort showed PFA, probably related to the fact that no anti‐Mi2 antibodies were found in them.^10^ Different from PFA, the PFN patients exhibited the most severe muscle weakness along with the highest serum CK levels, which could be explained by the highest score of necrotic fibers in this subgroup. Anti‐Mi2 and anti‐ARS antibodies (especially anti‐Jo‐1 antibodies) constituted the majority of MSAs in PFN subgroup, which was in line with the previous studies.^16^, ^17^, ^20^

The underlying pathomechanism behind different PF changes remained to be explored. In PFA patients, the marked expression of type I IFN‐inducible proteins preferentially located in perifascicular myofibers indicated the close relationship between type I IFN and PFA.^14^, ^15^ What's more, as proposed in both our present and previous work, PFA phenomena often occurred in fascicles adjacent to a wide perimysium.^32^ A recent study also reported typical sub‐/perifascial myofiber atrophy in eosinophilic fasciitis with upregulation of MHC‐I and MHC‐II, but without any signs of a type I IFN response or hypoxia mediated processes.^33^ Altogether, we supposed that damage of collagen and other structural protein in the perimysium or fascia might also contribute to the development of PFA, eventually leading to mechanical conduction disturbance and muscle weakness.^21^, ^34^ As we observed in this study, MAC deposition on PF capillaries was a prominent characteristic of PFA, which strengthened again the role of complement‐mediated capillary endothelial injury in DM‐PFA.^35^, ^36^ This opinion was also in line with the previous hypoxia theory concerning PFA, which showed that capillary MAC deposition caused focal microvascular and transverse vessel depletion, ultimately resulting in abnormal blood supply and hypoxia in the PF area.^12^, ^13^ Different from that in PFA, MAC mainly deposited on non‐necrotic sarcolemma of myofibers, especially along the PF side in PFN patients. This suggested that complement activation probably preferentially attacked the myofibers themselves in PFN individuals.^3^, ^16^ We also observed the most characteristic inflammatory pattern of lymphocyte infiltration in PFN subgroup: endomysial infiltration mainly alongside the PF region, which was probably associated with the perifascicular necrotic fibers.^20^, ^37^ With a similar follow‐up duration, we found the PFN patients, most of whom carrying anti‐Mi2 antibodies, were more likely to be drug‐free with complete recover. This was in line with several previous studies.^38^, ^39^ Liang et al.^39^ also found that 97.0% of the anti‐Mi2 positive patients showed clinical remission at follow‐up. Jiang et al.^38^ demonstrated that anti‐Mi2‐positive patients had a better survival rate than anti‐other‐DMSA‐positive patients and MSA‐negative patients. We proposed that the necrosis fibers were more likely to be replaced by regenerated myofiber and restore muscle strength due to good response to enough and regular immunosuppressive treatment. However, the regeneration capacity might be weaker for the atrophic and degenerative myofibers, thus might leave long‐lasting muscle weakness and treatment in PFA patients.

To the best of our knowledge, the group with isolated PF‐MHCn expression in IIMs was first proposed as an independent subgroup in the present study. Actually, MHCn expression was common in IIMs and other mimicking muscular disease, while the peri‐toward‐centrofascicular gradient pattern might still suggest a specific pathogenic role and an important significance for the diagnosis of IIM on muscle pathology.^6^, ^40^ The patients with isolated PF‐MHCn accounted for 22.0% (26/118) of the total patients with PF changes in our cohort. The classic clinical features, such as skin rashes and ILD, in PF‐MHCn patients were similar to those in the other two subgroups. However, nearly half of the patients with isolated PF‐MHCn expression were negative for MSAs, suggesting that these patients might have some undiscovered MSAs contributing to the unique pathological phenotype. Pathologically, although neither PFA nor PFN could be identified in the patients with isolated PF‐MHCn upregulation, scattered necrotic fibers in the centrofascicle were commonly seen, which might cause the high CK levels in these patients. Actually, this is consistent with a recent study which reported that 17% of the PM patients with necrotic myofibers showed PF‐MHC‐I expression.^6^ MxA and MAC expression were statistically rarer in this subgroup. This suggested that different cytokine and MAC pathways might contribute to different PF changes. Therefore, we would propose PF‐MHCn as an independent pathological subgroup, similar to PFA and PFN. Further intensive research regarding this subgroup is warranted in the future.

Using machine learning based tree classification algorithms, we finally confirmed that three different types of PF changes could be accurately predicted by specific clinico‐serological profiles, referring to anti‐Mi2 antibodies, MSAs‐negative, anti‐Jo‐1 and non‐Jo‐1 antibodies, serum CK levels, and MMT8. The distinguish of PFA and PFN subgroups could be effectively predicted by three variables (CK ≥ 1000 U/L, anti‐Mi2, and anti‐Jo‐1 antibodies) in the decision tree, of which the accuracy and AUCs were 87% and 0.872, respectively (decision tree in Stage 3). Herein, without invasive muscle biopsy, we could also accurately predict whether a newly diagnosed IIM patient would suffer from PFA or PFN, which were linked to different prognosis and treatment strategies. In contrast, the accuracy and AUCs of decision tree in Stage 2 to classify PF‐MHCn and “PFA/PFN” subgroups were 82.2% and 0.769 respectively, but the sensitivity was about 50%, which was unsatisfied. These results might suggest the unknown complexity of patients with PF‐MHCn.

## 5 LIMITATION

The present study has several limitations. First, as a single‐center retrospective cohort study, inevitable selection bias and data missing might occur during the patient enrollment process. Second, the MSAs detection by dot immunoassay was not reconfirmed by the gold criteria immunoprecipitation, which might result in false‐positive and false‐negative outcomes. However, the latter assay is not available for routine clinical use and only for research laboratories. Lastly, the prognosis analysis was not comprehensive due to the limited data based on outpatient records and the telephone follow‐up.

## 6 CONCLUSION

There are three different types of PF changes in IIM patients, each referring to different clinico‐serological characteristics, which suggests distinct underlying pathogenic mechanisms and the need for more precise treatment approaches for each type. A tree‐based three‐stage machine learning model established in our study could accurately predict whether a newly diagnosed IIM patient would develop pathological PF changes and specifically PFA, PFN, or PF‐MHCn.

## AUTHOR CONTRIBUTIONS

Zhang Lining: Conceptualization, Methodology, Data curation, Writing‐Original draft preparation. Fu Lijun: Methodology, Writing‐Original draft preparation. Zhang Guoyong, Ma Xiaotian, Zhao Dandan, Li Wei: Investigation, Resources. Hou Ying, Dai Tingjun, Shu Qiang: Validation. Yan Chuanzhu, Zhao Bing: Conceptualization, Methodology, Writing‐Reviewing and Editing and Funding acquisition.

## CONFLICT OF INTEREST STATEMENT

The authors reported no disclosures relevant to the manuscript.

## Supporting information


Appendix S1

Appendix S2

## Data Availability

Unpublished data related to this article are available on request from the authors.
