# Supplementary material for: Clinico‐sero‐pathological profiles and risk prediction model of idiopathic inflammatory myopathy (IIM) patients with different perifascicular changes
Source: CNS Neurosci Ther. 2024 Aug 4;30(8):e14882. doi: 10.1111/cns.14882 (PMC11298199; doi:10.1111/cns.14882)
Supplement: Supplementary file 1 — Appendix S1 [file CNS-30-e14882-s002.docx]

1.1 Logistic regression-decision tree (LR-DT) model

As shown in **Supplementary Figure S1**, the learning task of the first stage is to classify whether an IIM patient will suffer from muscular PF changes based on the clinic-serological features (Stage 1). At the second stage, the decision tree was applied to classify PFA/PFN from PF-MHCn (Stage 2). At the final stage, the decision tree could distinguish whether the patient would present with PFA or PFN (Stage 3). In the three prediction trees, the featured selection was decided by priori knowledge and a specific logistic regression model.


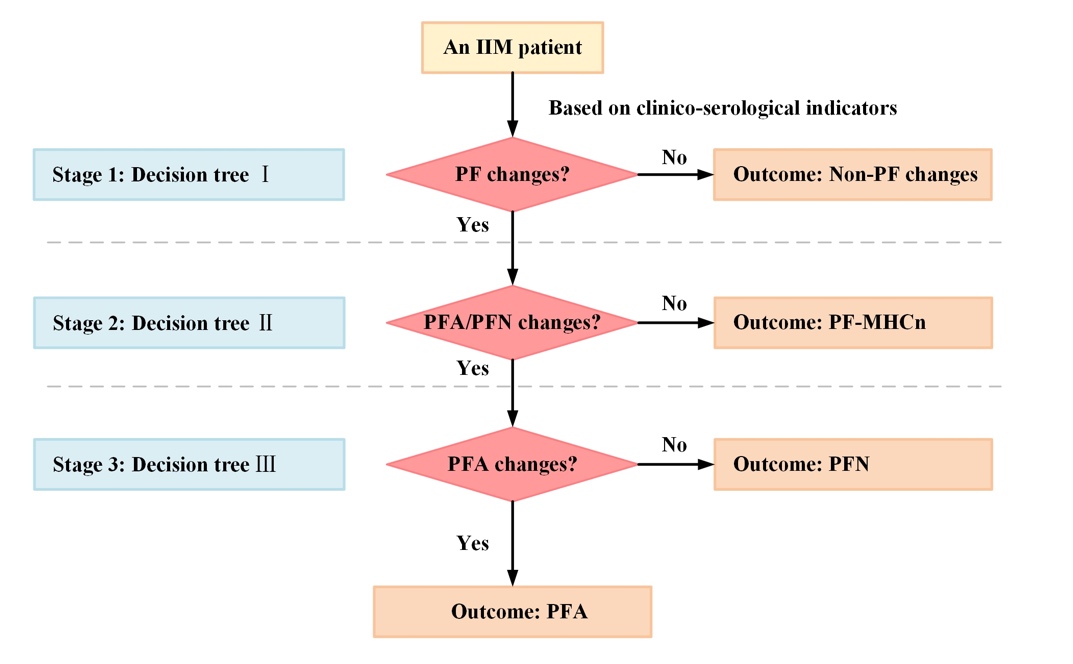


**Supplementary Figure S1.** A three-stage prediction process for predicting PF change subtypes.

Abbreviation: IIM=idiopathic inflammatory myopathy; PF=perifascicular area; PFA=perifascicular atrophy; PFN= perifascicular necrosis; PF-MHCn= perifascicular enhancement of MHC-I and/or MHC-II; MHC= major histocompatibility complex.

1.2 Main outcome measures

The overall discriminant performances of the proposed model were assessed by accuracy, specificity, sensitivity, F1 score and the area under the receiver operating characteristic (ROC) curve (AUC). Based on a confusion matrix (**Supplementary Table S1**), these indicators except AUC are calculated as Equation (1).

**Supplementary Table S1.** An illustration of confusion matrix associated with muscular PF changes in step 1.

|  |  | Model predictions | |
| --- | --- | --- | --- |
|  |  | PF changes | Non-PF changes |
| Actual categories | PF changes | True Positives (TP) | False Negatives (FN) |
|  | Non-PF changes | False Positives (FP) | True Negatives (TN) |

$$\begin{aligned} \begin{matrix} \begin{matrix} accuracy=\frac{TP+TN}{TP+NP+TN+FN} \\ specificity=\frac{TP}{TP+FP} \end{matrix} \\ \begin{matrix} sensitivity=\frac{TP}{TP+FN} \\ F1score=\frac{2specificity\times sensitivity}{specificity+sensitivity} \end{matrix} \end{matrix}\#\left( 1 \right) \end{aligned}$$

Reference

[1] Wang Y, Wang D, Ye X, et al. A tree ensemble-based two-stage model for advanced-stage colorectal cancer survival prediction[J]. Information Sciences, 2019, 474: 106-124.
