# Supplementary material for: Clinico‐sero‐pathological profiles and risk prediction model of idiopathic inflammatory myopathy (IIM) patients with different perifascicular changes
Source: CNS Neurosci Ther. 2024 Aug 4;30(8):e14882. doi: 10.1111/cns.14882 (PMC11298199; doi:10.1111/cns.14882)
Supplement: Supplementary file 2 — Appendix S2 [file CNS-30-e14882-s001.docx]

### Supplementary Table S2. Clinical and histopathological features between IIM patients with and without muscle perifascicular changes.

|  | Total  (n=231) | PF group  (n=118) | Non-PF group  (n=113) | *P*-value |
| --- | --- | --- | --- | --- |
| Female, n(%) | 142(61.5) | 73(61.9) | 69(61.1) | 0.900 |
| Age of onset, median(IQR)  years | 50(36,64) | 50(34,64) | 51(38,63) | 0.352 |
| Juvenile^†^, n(%) | 31(13.4) | 20(16.9) | 11(9.7) | 0.108 |
| Disease duration^#^, median(IQR) months | 4(2,12) | 4(2,11) | 3(1,12) | 0.414 |
| Skin involvement, n(%) | 146(63.2) | 87(73.7) | 59(52.2) | 0.001^*^ |
| Gottron’s sign/papules | 61(26.4) | 35(29.7) | 26(23.0) | 0.252 |
| Heliotrope rash | 55(23.8) | 36(30.5) | 19(16.8) | 0.015^*^ |
| Muscle weakness, n(%) | 205(88.7) | 108(91.5) | 97(85.8) | 0.172 |
| MMT8, median(IQR) | 68(58,75) | 66(56,74) | 70(60,76) | 0.021^*^ |
| Myalgia, n(%) | 120(51.9) | 59(50.0) | 61(54.0) | 0.545 |
| Arthralgia, n(%) | 61(26.4) | 30(25.4) | 31(27.4) | 0.729 |
| Dysphagia, n(%) | 84(36.4) | 47(39.8) | 37(32.7) | 0.263 |
| ILD^‡^, n(%) | 84(37.8) | 48(42.5) | 36(33.0) | 0.147 |
| Malignancy^§^, n(%) | 23/226(10.2) | 12(10.2) | 11/108(10.2) | 0.951 |
| CK at biopsy, median(IQR) U/L | 1354(279,3838) | 1485(303,3666) | 1101(210,4163) | 0.526 |
| MSAs^¶^, n(%) |  |  |  |  |
| DMSA | 101(45.9) | 68(60.2) | 33(30.8) | ＜0.001^*^ |
| Anti-TIF1γ | 28(12.7) | 17(15.0) | 11(10.3) | 0.289 |
| Anti-NXP2 | 28(12.7) | 16(14.2) | 12(11.2) | 0.513 |
| Anti-Mi2 | 29(13.2) | 25(22.1) | 4(3.7) | ＜0.001^*^ |
| Anti-MDA5 | 15(6.8) | 9(8.0) | 6(5.6) | 0.488 |
| Anti-SAE | 2(0.9) | 2(1.8) | 0(0) | 0.498 |
| Anti-ARS | 49(22.3) | 21(18.6) | 28(26.2) | 0.177 |
| Anti-Jo1 | 21(9.5) | 13(11.5) | 8(7.5) | 0.310 |
| Anti-PL7 | 6(2.7) | 1(0.9) | 5(4.7) | 0.111 |
| Anti-PL12 | 3(1.4) | 2(1.8) | 1(0.9) | ＞0.999 |
| Anti-EJ | 6(2.7) | 1(0.9) | 5(4.7) | 0.111 |
| Anti-OJ | 6(2.7) | 1(0.9) | 5(4.7) | 0.111 |
| Anti-Ha | 8(3.6) | 3(2.7) | 5(4.7) | 0.489 |
| MSAs-negative | 73(33.2) | 26(23.0) | 47(43.9) | ＜0.001^*^ |

Abbreviation: IIM=idiopathic inflammatory myopathy; IQR=interquartile range；MMT=manual muscle testing; ILD=interstitial lung disease; CK=creatine kinase; MSAs=myositis-specific autoantibodies; DMSA=dermatomyositis-specific autoantibodies; ARS=anti-aminoacyl tRNA synthetase; PF=perifascicular area. ^†^Age≤18 years; ^#^ The time from the first myositis symptom to the diagnosis of IIM; ^‡^N=222 (113+109); ^§^N=226 (118+108); ^¶^N=220 (113+107).

### Supplementary Table S3. Histopathological differences between IIM patients with and without muscle perifascicular changes.

|  | Total  (n=231) | PF group  (n=118) | Non-PF group  (n=113) | *P*-value |
| --- | --- | --- | --- | --- |
| Myofiber abnormalities |  |  |  |  |
| Myofiber necrosis score, median(IQR) | 1(0,2) | 1(0,2) | 1(0,2) | 0.982 |
| Myofiber necrosis, n(%) | 160(69.3) | 81(68.6) | 79(69.9) | 0.835 |
| Punch-out fibers, n(%) | 62(26.8) | 55(46.6) | 7(6.2) | ＜0.001^*^ |
| MHC-I expression, n(%) | 221(95.7) | 118(100) | 103(91.2) | 0.003^*^ |
| MHC-II expression^†^, n(%) | 132(57.6) | 84(72.4) | 48(42.5) | ＜0.001^*^ |
| MxA expression^†^, n(%) | 103(45.0) | 78(67.2) | 25(22.1) | ＜0.001^*^ |
| MAC deposition^†^, n(%) |  | | | |
| On non-necrotic sarcolemma | 109(47.6) | 60(51.7) | 49(43.4) | 0.205 |
| On capillaries | 101(44.1) | 68(58.6) | 33(29.2) | ＜0.001^*^ |
| Inflammatory infiltration score^‡^, median(IQR) | | | | |
| Total score | 3(0,5) | 2(1,5) | 3(0,4) | 0.300 |
| Perimysial score | 1(0,2) | 1(0,2) | 1(0,2) | 0.642 |
| Endomysial score | 1(0,2) | 1(0,2) | 1(0,2) | 0.706 |
| Perivascular score | 1(0,1) | 1(0,2) | 1(0,1) | 0.243 |
| Histochemical staining, n(%) |  |  |  |  |
| Decreased/absent COX activity | 128(55.4) | 78(66.1) | 50(44.2) | 0.001^*^ |
| Blue fiber on S/C double staining | 98(42.4) | 56(47.5) | 42(37.2) | 0.114 |

Abbreviation: IIM=idiopathic inflammatory myopathy; IQR=interquartile range；PF=perifascicular area; MHC= major histocompatibility complex class; MxA= myxovirus resistance protein; MAC=membrane attack complex; COX=cytochrome oxidase; SDH= succinate dehydrogenase. ^†^N=229 (116+113); ^‡^Total score of CD3 and CD20 lymphocyte: ＜4cells/20HPF=0；4~10(1 cluster)/20HPF=1；≥2 clusters or＞20cells/20HPF=2.

### Supplementary Table S4. Classification performance of the proposed tree-stage LR-DT models.

| Stage |  | Step1 | |  | Step2 | |  | Step3 | |
| --- | --- | --- | --- | --- | --- | --- | --- | --- | --- |
| dependent variable |  | PF changes | |  | PFA/PFN | |  | PFA | |
| Model |  | LR1 | Model Ⅰ |  | LR2 | Model Ⅱ |  | LR3 | Model Ⅲ |
| Accuracy |  | 0.668 | 0.682 |  | 0.772 | 0.822 |  | 0.904 | 0.870 |
|  |  | (0.100) | (0.104) |  | (0.082) | (0.112) |  | (0.109) | (0.108) |
| Precision |  | 0.689 | 0.732 |  | 0.790 | 0.838 |  | 0.920 | 0.858 |
|  |  | (0.114) | (0.134) |  | (0.065) | (0.107) |  | (0.103) | (0.131) |
| Recall |  | 0.672 | 0.699 |  | 0.967 | 0.97 |  | 0.923 | 0.960 |
|  |  | (0.135) | (0.170) |  | (0.071) | (0.071) |  | (0.130) | (0.080) |
| F1 score |  | 0.673 | 0.665 |  | 0.867 | 0.894 |  | 0.915 | 0.898 |
|  |  | (0.102) | (0.119) |  | (0.053) | (0.067) |  | (0.099) | (0.080) |
| AUC |  | 0.703 | 0.708 |  | 0.674 | 0.769 |  | 0.949 | 0.872 |
|  |  | (0.094) | (0.088) |  | (0.191) | (0.214) |  | (0.068) | (0.132) |
| N of observations |  | 220 | 220 |  | 118 | 118 |  | 92 | 92 |

Note: LR = logistic regression, three LR models were constructed by all 13 clinic-serological profiles. Model LR1 eliminated 11 samples with clinical indicator missing rates exceeding 60%. The results for the mean and standard deviation (in brackets) are obtained within 10-fold cross validation.


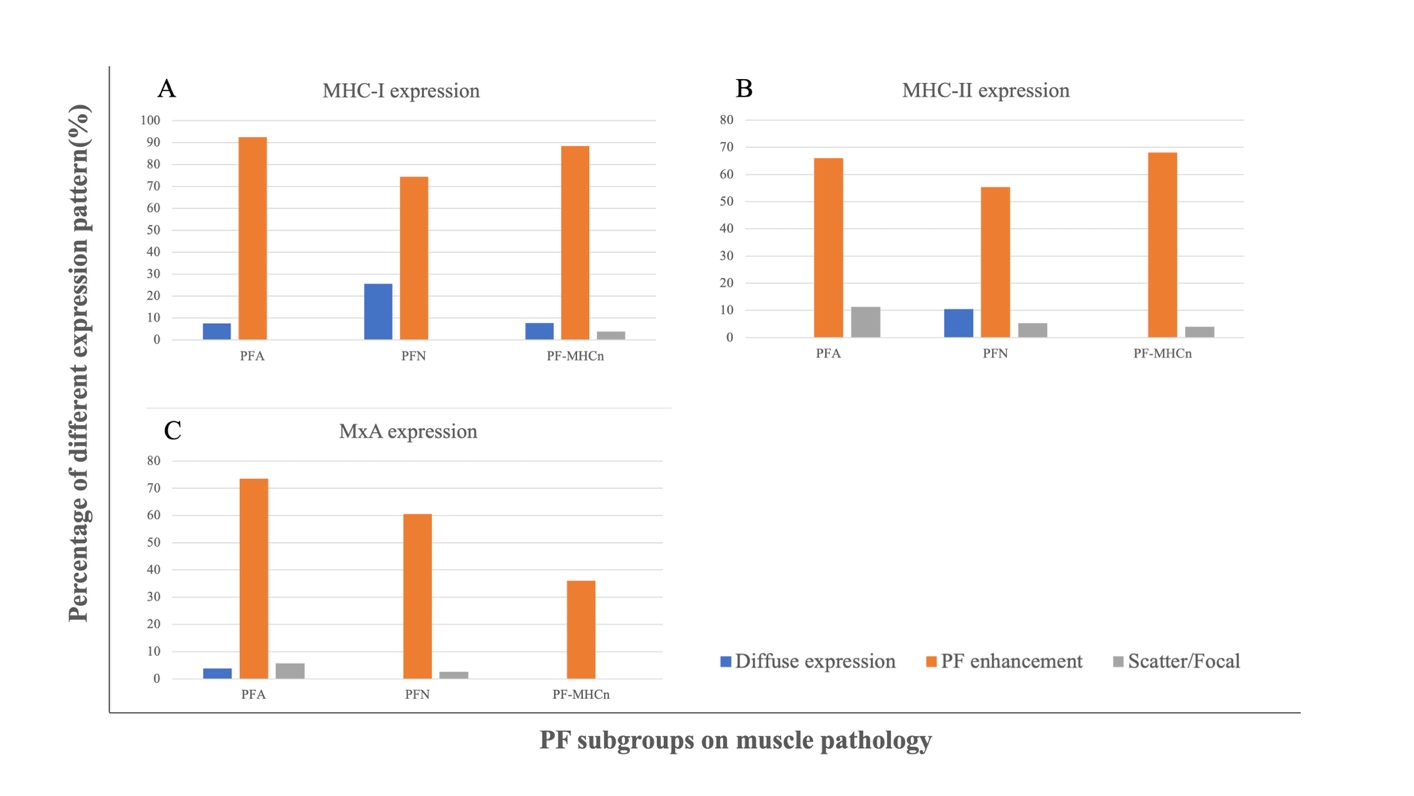


### Supplementary Figure S2. Percentages of 3 expression patterns of MHC-I(A), MHC-II(B) and MxA(C) in IIM patients. Abbreviation: PFA=perifascicular atrophy; PFN= perifascicular necrosis; PF-MHCn= perifascicular enhancement of MHC-I and/or MHC-II; PF=perifascicular area; MHC= major histocompatibility complex class; MxA= myxovirus resistance protein.
